# Does Slow and Steady Win the Race? Rates of Antipsychotic Discontinuation, Antipsychotic Dose, and Risk of Psychotic Relapse

**DOI:** 10.1093/schbul/sbad139

**Published:** 2023-10-05

**Authors:** Robert A McCutcheon, David Taylor, Jose Rubio, Joseph Nour, Toby Pillinger, Robin M Murray, Sameer Jauhar

**Affiliations:** Department of Psychiatry, University of Oxford, Oxford, UK; Oxford Health NHS Foundation Trust, Oxford, UK; Department of Psychosis Studies, Institute of Psychiatry, Psychology and Neuroscience, London, UK; South London and Maudsley NHS Foundation Trust, London, UK; Department of Psychiatry, Northwell Health, The Zucker Hillside Hospital, Glen Oaks, NY, USA; East London NHS Foundation Trust, London, UK; Department of Psychosis Studies, Institute of Psychiatry, Psychology and Neuroscience, London, UK; South London and Maudsley NHS Foundation Trust, London, UK; Department of Psychosis Studies, Institute of Psychiatry, Psychology and Neuroscience, London, UK; South London and Maudsley NHS Foundation Trust, London, UK; Department of Psychological Medicine, Institute of Psychiatry, Psychology and Neuroscience, London, UK

**Keywords:** withdrawal, schizophrenia, psychosis, treatment

## Abstract

**Background:**

Antipsychotics are recommended for prevention of relapse in schizophrenia. It is unclear whether increased risk of relapse following antipsychotic discontinuation is predominantly associated with an absolute magnitude of dose reduction or rate of antipsychotic reduction. Establishing the responsible mechanism is important because prolonged withdrawal schedules have been suggested to reduce risk of relapse.

**Study Design:**

Individual patient data from antipsychotic discontinuation studies were obtained. We estimated the occupancy of receptors over time using half-lives and median effective dose ED_50_ values obtained from pharmacokinetic and receptor occupancy studies. Hazard ratios for relapse events were calculated using Cox proportional hazards models to assess the influence of formulation (oral, 1-monthly, and 3-monthly injections). The change in hazard ratio over time was estimated, and the effect of time-varying covariates was calculated, including rate of occupancy reduction and absolute receptor occupancy.

**Study Results:**

Five studies including 1388 participants with schizophrenia were identified (*k* = 2: oral, *k* = 2: 1-monthly injection, *k* = 1: 3-monthly injection). Withdrawal of long-acting injectable medication did not lead to a lower hazard ratio compared with withdrawal of oral medication, and this included the period immediately following randomization. Hazard ratios were not associated with the rate of decline of receptor occupancy; however, they were associated with reduced absolute occupancy in trials of long-acting injections (*P* = .038).

**Conclusions:**

Antipsychotic discontinuation is associated with an increased risk of psychotic relapse, related to receptor occupancy. Although relapse does not appear to be related to the rate of discontinuation, gradual discontinuation strategies may allow for easier antipsychotic reinstatement in case of symptomatic worsening.

## Introduction

Schizophrenia is a relapsing remitting disorder with relapse rates acknowledged to be as high as 85% in cohorts pre-dating the modern psychopharmacology era.^1^ Relapses after the initial episode are associated with functional deterioration, suicidal behavior, and increased costs.^2–4^ Schizophrenia is associated with increased striatal dopamine signaling and dopamine antagonism is the mainstay of its pharmacological treatment.^5,6^ The value of dopamine antagonists in preventing relapse in schizophrenia is one of their most well-established benefits.^7–9^ Antipsychotic treatment is, however, associated with significant side effects and, as a result, both patients and clinicians may wish to consider dose reduction or discontinuation when symptoms have been stable for a sustained period.^10–12^

Antipsychotic discontinuation is strongly associated with an increased risk of relapse.^8,13,14^ Several studies have suggested that, although antipsychotic dosage can be reduced in those with stable symptoms, risk of relapse increases when dosages fall below the recommended treatment range.^15,16^ These findings are consistent with a model in which a minimal threshold of dopamine receptor occupancy is required to reduce the risk of relapse.^17^

In addition, some authors have argued that not only a minimal threshold of consistent dopamine receptor occupancy, but also rapid changes in occupancy may be a key factor in determining the risk of relapse, with abrupt cessation more likely to precipitate relapse than a gradual discontinuation.^12,18^ Recent recommendations regarding medication reduction have suggested a prolonged “hyperbolic” strategy in which the magnitude of dose reduction decelerates over time may be particularly effective in ameliorating the risk of relapse.^12^ This approach suggests that reducing the rate of antipsychotic discontinuation may reduce the risk of relapse. This, however, remains controversial, since meta-analytic data converge in not finding large differences in relapse risk after discontinuation between abrupt and progressive tapers.^13,19^

Thus, although the data are quite compelling in indicating that relapse risk is mediated by pharmacodynamic factors, and ultimately related to dopamine receptor occupancy, the details about the specific role of absolute occupancy and rate of antipsychotic reduction are not yet well understood. Providing clarity on this could have important implications both for our understanding of the pathoetiology of relapse and for designing discontinuation schemes.

We aim to examine the role of absolute reductions in receptor occupancy and rate of receptor occupancy by analyzing individual-level data from relapse prevention trials of three antipsychotic formulations with widely varying half-lives. If the rate of withdrawal is an important factor in determining relapse, then relapse risk should be greater during rapid reductions in receptor occupancy. If, however, it is primarily the absolute level of occupancy that is the causal factor, then relapse risk should not differ significantly between formulations, and should relate to occupancy.

## Methods

### Data Extraction

We searched the Yale University Open Data Access (YODA) database^20^ to identify relapse prevention trials of antipsychotic medications in individuals aged 18–65 with schizophrenia or schizoaffective disorder. We included double-blind, placebo-controlled randomized controlled trials in which individuals were treated with antipsychotics for over 3 months and clinically stabilized prior to randomization to placebo or continued active treatment. We identified five trials that compared oral, 1-monthly injectable, and 3-monthly injectable formulations of paliperidone to placebo. For each individual, we calculated the time to psychosis relapse (defined as per original study criteria) or censoring.

### Receptor Occupancy

In all trials, the initial stabilization period involved optimizing dosing to maximize efficacy and tolerability. For the main analysis, we therefore assumed that individuals had a trough D2 receptor occupancy level (ie, the occupancy level at the point of randomization) of 80%, given that this is considered to be a level at which efficacy is maximized without inducing severe extra pyramidal side effects.^21–23^

This occupancy level of 80% was then converted to a trough paliperidone plasma level based on a striatal median effective dose (ED_50_) of 2.38 mg/day.^24^ The peak plasma level was then calculated based on the trough level, the time to peak level (5 days for 1-monthly and 28 days for 3-monthly injections),^25,26^ and the dosing interval (30 days for 1-monthly and 90 days for 3-monthly), see Supplementary File for full details of calculation. We did not investigate the role of receptor occupancies in studies using oral medication as occupancy reaches 0% too rapidly to allow for time-varying analyses.^27^

Plasma levels were assumed to rise linearly following injection until reaching the peak at which point we calculated a first-order decay until the next dosing (with a half-life of 37 days for 1-monthly and 111.5 days for 3-monthly injections).^28,29^ For individuals randomized to placebo, a first-order decay was assumed to occur from the trough level at the point of randomization.

The estimated plasma levels were translated to estimated receptor occupancy levels according to the *E*_max_ equation of the form:


$$\begin{array}{l} Receptor\;occupancy=maximal\;occupancy\times dose/\left(dose+ED_{50}\right)\; \end{array}$$

**Fig. 1.**
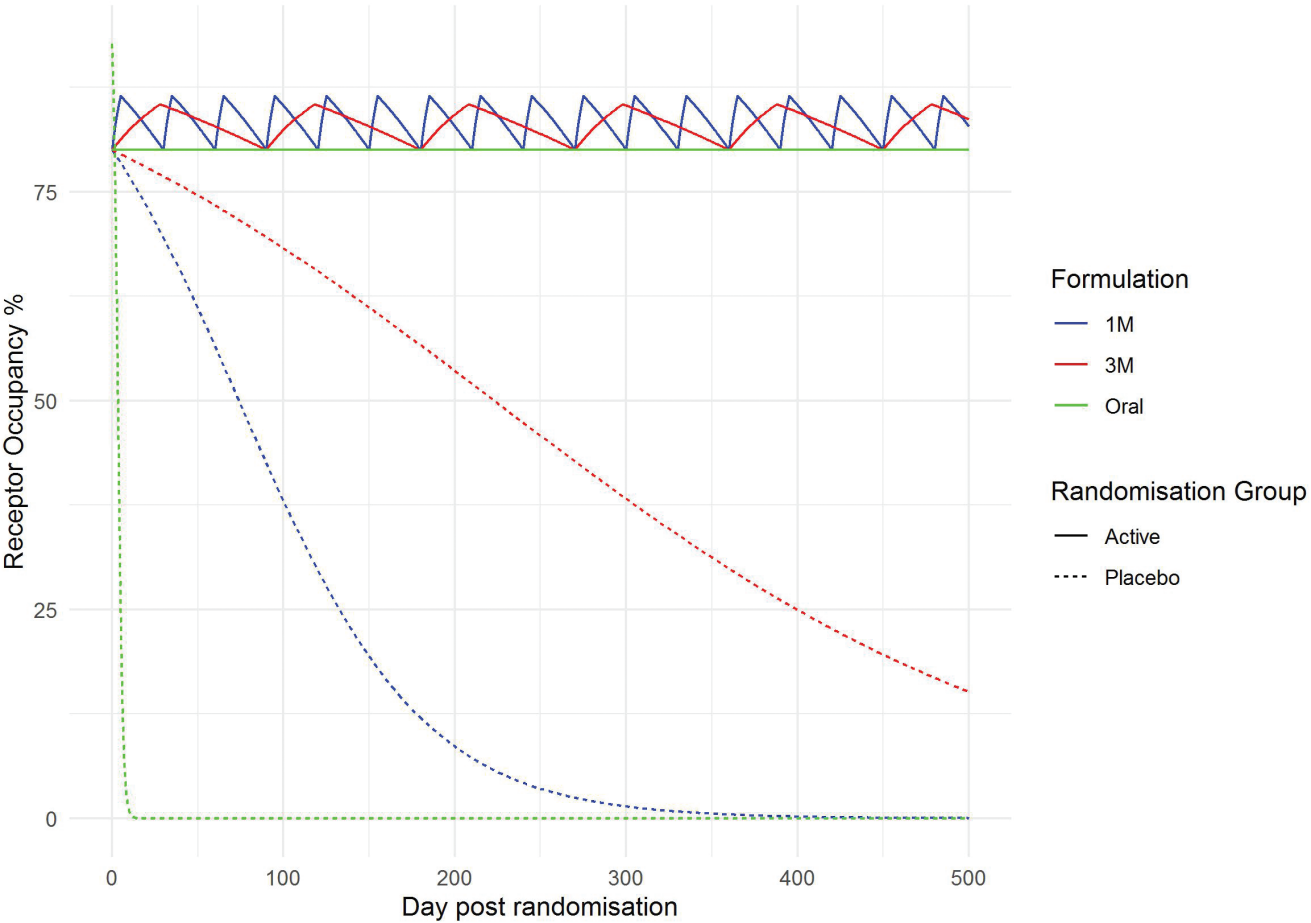
Receptor occupancy levels over time. Illustration of the relationship between duration of antipsychotic cessation and receptor occupancy for three formulations of paliperidone. Active oral treatment appears constant as plot only reports a daily average.

The value of ED_50_ was taken as 2.38 mg/day with maximal occupancy at 100% based on the findings from receptor occupancy studies.^24^ The resulting occupancy levels are illustrated in figure 1, for the active treatment arm receptor occupancies range between 80% and 86% for 1-monthly injections, and 80% and 85% for 3-monthly injections.

We also performed sensitivity analyses for all subsequent analyses in which the trough occupancy level was assumed to be 75% or 85%.

### Statistical Analysis

We performed five analyses. First, we assessed whether relapse hazard ratios differed across trials between paliperidone formulations, that is, whether formulations with longer half-lives were more protective against relapse than those with shorter half-lives. Second, we assessed whether hazard ratios differed within trials over time, that is, whether hazard ratios were greatest near the study start (which might be expected if sudden withdrawal increases risk of relapse) or increased with time (which might be expected if duration of time without receptor blockade is a risk factor for relapse). Third, we investigated within trials whether periods during which the rate of decline of receptor occupancy was greater were associated with increased hazard ratios. Fourth, we investigated whether within trials the absolute value of receptor occupancy was associated with hazard ratios. In all analyses, statistical significance was defined as *P* < .05 and specific details are as follows.

i) Effect of formulation on relapse risk

We investigated whether the risk of relapse varied according to formulation. Cox proportional hazard models were fit to each study independently using the *Survival* package^30^ with randomization group (placebo vs active treatment) as the sole predictor. A Wald-type test was then performed with formulation as the moderating factor to determine whether formulation was predictive of hazard ratio across studies.

ii) Effect of time since randomization on relapse risk

We examined within trials whether the hazard ratio decreased with time to test whether relapses were particularly associated with the early weeks of a trial in oral discontinuation studies. We employed the approach implemented in the *casebase* package which allows for hazard ratios to vary over time, in contrast to the Cox model in which these are fixed. We examined whether there was an interaction between the randomization group and time in terms of relapse rates^31,32^; if a significant interaction exists, it means that the hazard ratio is not constant over time.

iii) Effect of rate of change of receptor occupancy on relapse risk

We then investigated whether the rate of change of receptor occupancy in trials of long-acting injectables was associated with relapse risk. The daily rate of change of receptor occupancy was defined as the first derivative of the receptor occupancies calculated above. For each study, proportional hazard models were fit with the randomization group and the daily change as predictors. Coefficients were then combined within formulations via random effects meta-analysis implemented in *metafor*.^33^

iv) Effect of absolute receptor occupancy on relapse risk

We investigated whether absolute receptor occupancy was associated with relapse risk. For each study, proportional hazard models were fit with randomization group as a constant covariate and daily estimated occupancy (as illustrated in figure 1) as a time-varying covariate.^34^ As before, coefficients were then combined within formulations via random-effects meta-analysis.

## Results

### Included Studies

Five eligible studies were identified, encompassing 1388 participants (table 1). All compared paliperidone to placebo, two used an oral formulation, two used a 1-monthly long-acting injection, and one used a 3-monthly long-acting injection.

**Table 1.** Characteristics of included studies

Study	YODA code	NCT code	Study design	Interventions	Participant characteristics
Berwaerts et al^35^	PSY-3012	NCT01529515	Double-blind; parallel; up to 16 months	3-monthly LAI (*n* = 159) Placebo (*n* = 144)	Schizophrenia (78% male, mean age 37.8 years)
Fu et al^36^	SCA-3004	NCT01193153	Double-blind; parallel; up to 16 months	1-monthly LAI (*n* = 164) Placebo (*n* = 170)	Schizophrenia/schizoaffective disorder (51% male, mean age 38.6 years)
Hough et al^37^	PSY-3001	NCT00111189	Double-blind; parallel; up to 16 months	1-monthly LAI (*n* = 205) Placebo (*n* = 203)	Schizophrenia (54% male, mean age 42.8 years)
Kramer et al^38^	SCH-301	NCT00086320	Double-blind; parallel up to 12 months	Oral (*n* = 105) Placebo (*n* = 102)	Schizophrenia (59% male, mean age 38.2 years)
Rui et al^39^	SCH-3041	NCT01662310	Double-blind; parallel up to 14 months	Oral (*n* = 65) Placebo (*n* = 71)	Schizophrenia (40% male mean age 31.6 years)

*Note*: LAI, long-acting injection.

i) Effect of formulation on relapse risk

**Fig. 2.**
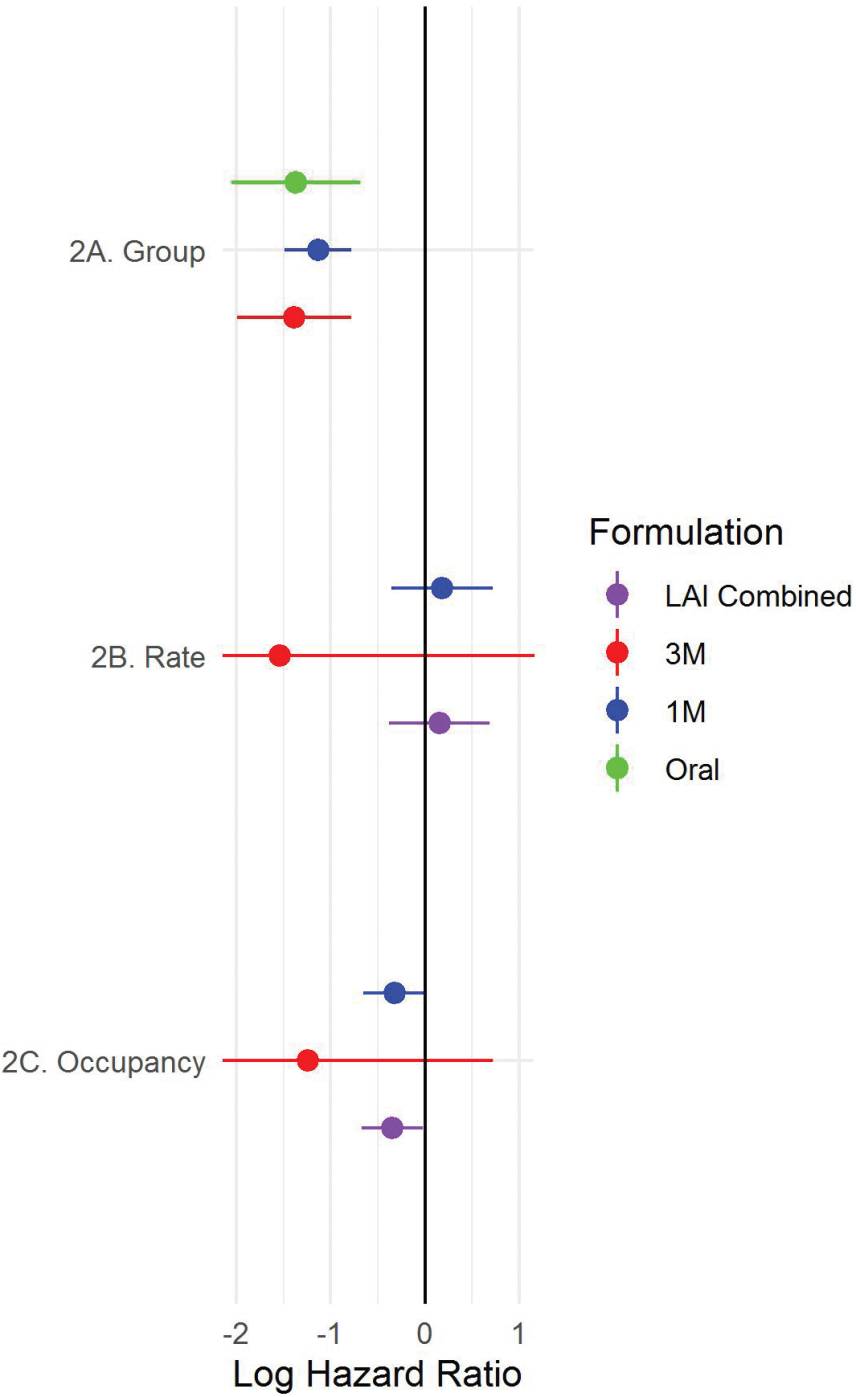
Hazard ratios relating to randomization group, receptor occupancy, and rate of occupancy change. (A) For the “Group” set of points, a negative log hazard ratio represents a reduced risk of relapse for active compared to placebo treatment. All formulations show a similar reduction in relapse risk compared to placebo. (B) For the “Rate” set of points, a negative log hazard ratio indicates that a slower decline in receptor occupancy within a trial is associated with a reduced risk of relapse. The observed positive value for the LAI combined analysis indicates that periods in which occupancy is most rapidly declining (ie, the initial months of a trial) are non-significantly associated with a reduced risk of relapse compared to placebo (*P* = .57). (C) For the “Occupancy” set of points, a negative log hazard ratio indicates that higher receptor occupancy is associated with a reduced risk of relapse. The observed negative value for the LAI combined analysis indicates that the risk of relapse relative to placebo increases as receptor occupancy falls. Horizontal bars represent the 95% confidence interval.

For all formulations, randomization to active treatment was associated with a reduced risk of relapse (*P* < .001, figure 2A). The hazard ratio did not differ between formulations (*P* > .4 for all pairwise comparisons).

ii) Effect of time since randomization on relapse risk

**Fig. 3.**
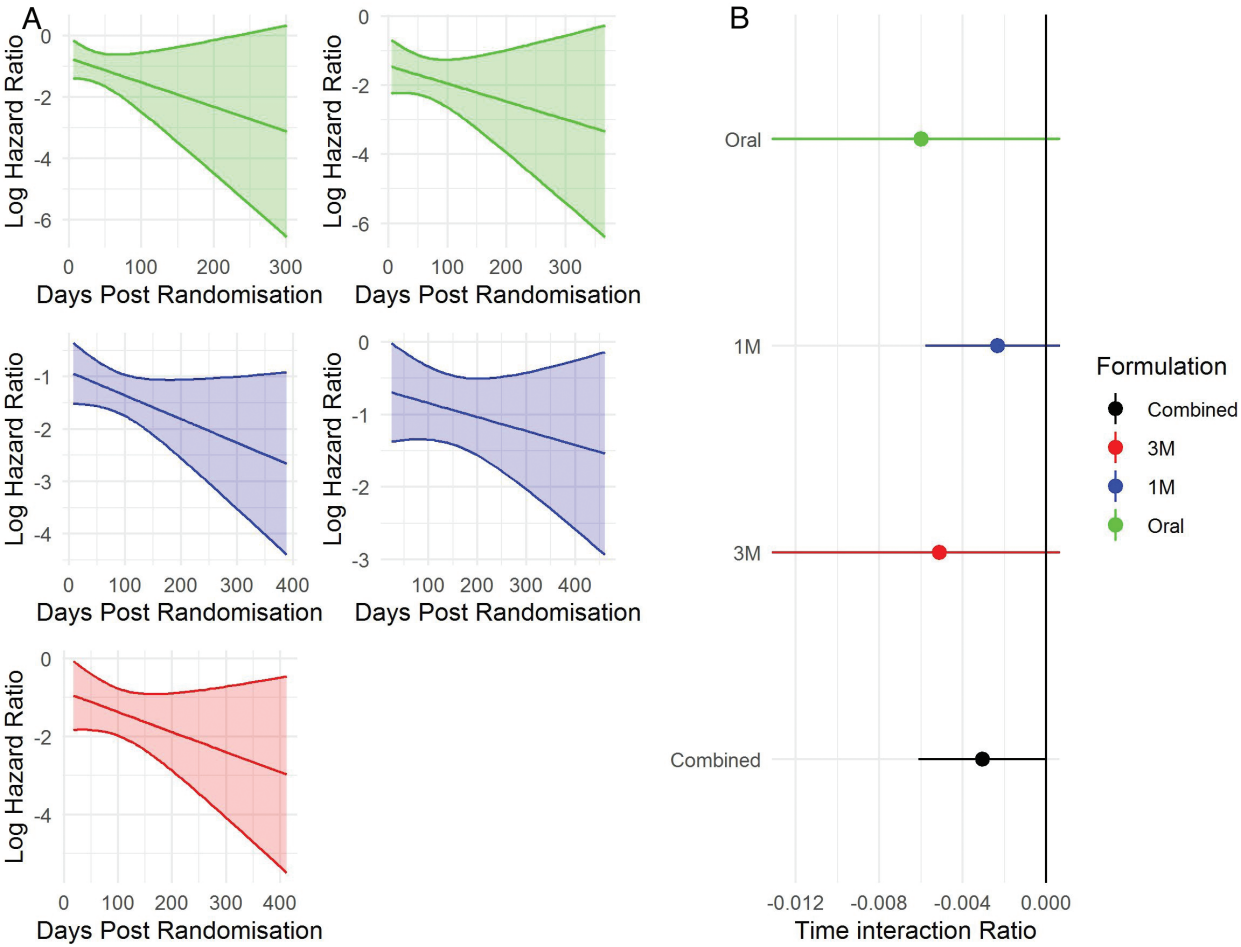
Relationship between hazard ratio and time. (A) In all trials, a negative log hazard ratio means that the risk of relapse is greater in the active treatment arm compared to the placebo arm, in all trials the relative protective effect of active treatment increases with time since randomization. (B) This interaction with time is statistically significant when all trials are combined (*P* = .04).

We examined whether hazard ratios varied by time post-randomization. If the hazard ratio was greater at the start of the trial and this was accentuated for trials using an oral formulation, it would imply that the rapid withdrawal of oral medication may contribute to the risk of relapse. For all formulations, the converse was seen in that the protective effect of active treatment increased with time (figure 3A) and this was statistically significant when all trials were meta-analytically combined (figure 3B, interaction ratio estimate −0.003, SE = 0.0015, *P* = .04). The time interaction ratio did not differ significantly between oral and long-acting injection trials (*P* = .52, figure 3B).

iii) Effect of rate of change of receptor occupancy on relapse risk

We examined the rate of reduction in receptor occupancy in trials of long-acting injections. Faster occupancy reduction was not significantly associated with relapse, and periods of faster reduction were not associated with a reduced risk of relapse (*P* = .57) (figure 2B).

iv) Effect of absolute receptor occupancy on relapse risk

We examined trials of long-acting injectables to determine whether estimated receptor occupancy was associated with the risk of relapse. We found that lower receptor occupancy levels were associated with an increased risk of relapse (estimate = −0.34, SE = 0.17, *P* = .037) (figure 2C).

Sensitivity analyses using different initial receptor occupancies demonstrated similar results (see Supplementary File)

## Discussion

In an analysis of 1388 individuals with schizophrenia from five randomized controlled trials, we found that the risk of relapse in those discontinuing antipsychotic treatment relative to continued treatment does not vary between formulations, is not frontloaded during the period immediately following abrupt oral antipsychotic discontinuation, and in trials of long-acting injectables is not associated with the rate of receptor occupancy decline. Instead, we find that the overall level of receptor occupancy is associated with the risk of relapse. These results suggest that extended withdrawal schedules may not significantly reduce the risk of relapse following antipsychotic discontinuation, and apparent benefits in reducing relapse may primarily relate to the extended period in which individuals receive dopamine receptor blockade. These findings also support the hypothesis that relapses observed in clinical trials are a distinct phenomenon from acute withdrawal symptoms.

To the best of our knowledge, this is the first study to explicitly examine how the relative risk of relapse varies over the time courses of discontinuation studies of different formulations and to investigate the relationship between the rate of receptor occupancy change and the risk of relapse. Our findings are broadly consistent with recent meta-analyses and observational studies that have demonstrated that the risk of relapse increases when an antipsychotic dose is reduced below recommended levels and that there is no difference in relapse rates following abrupt and gradual discontinuation.^13,15,16,40^ One of these meta-analyses did find that slower dose reduction (in reduction as opposed to discontinuation studies) was associated with a lower risk of relapse.^40^ This finding was complicated, however, by the fact that slow reduction studies often still used therapeutic doses of antipsychotics at the study end, and it was shown that dropping below a threshold of 5 mg haloperidol equivalents was associated with relapse, consistent with our findings.^40^ Prolonged dose-withdrawal schedules may simply extend the time taken for the therapeutic threshold to be crossed and so relapse only emerges at a later time.

Previous analyses, using overlapping samples with the current paper, have, however, reported a reduced risk of relapse in individuals withdrawing from formulations with a longer half-life.^41–43^ For instance, in a previous analysis, we found a lower risk of relapse following discontinuation of long-acting injectables compared with discontinuation of oral antipsychotics.^42^ The discrepancy with the current findings reflects the fact the earlier analyses examined raw relapse rates across different trials as opposed to the risk relative to the continued active treatment arm. Analyzing single arms of a trial in isolation, however, is inconsistent with the aim of clinical trials to isolate the effects of treatment.^44^ Trial-level factors must be accounted for, given that they correlate perfectly with formulation and may significantly influence relapse rates. If one examines raw relapse rates as opposed to the hazard ratio relative to a continued treatment arm, any conclusions reached may relate to specifics of individual trials, rather than more general points regarding formulation. Trial-level factors can lead to greater relapse rates in both the placebo *and* continued active treatment arms of oral trials, as discussed further below. Meta-analyses where relapse rates have been defined relative to a placebo comparator, thereby controlling for these trial-level effects, are consistent with the current findings, with relative risk of withdrawal following discontinuation of a long-acting injection not differing from the risk following oral discontinuation.^45^ This lack of difference in hazard ratios between oral and long-acting injectable antipsychotics contrasts with what is observed in observational studies.^46^ This is likely because clinical trials are less likely to recruit nonadherent participants, the population for whom injectable formulations are potentially more effective than oral formulations.

In the oral discontinuation trials, many relapses occur in both placebo and active arms near the start of the trial. This may relate to the incentive that triallists have to ensure that positive and negative syndrome scale (PANSS) scores remain below the eligibility threshold in the pre-randomization phase,^47^ which is subsequently relaxed following randomization. The reason why such a precipitous decline is not seen in trials of injectable formulations may relate to significantly longer pre-randomization phases (mean of 29 weeks compared with 14 weeks in oral trials), making it more challenging to erroneously suppress PANSS scores and the fact that longer stabilization period means that individuals with a high propensity for relapse are more likely to have relapsed and been excluded from the trial prior to the randomization point. These factors mean that it is vital that relapse rates are compared between placebo and active arms rather than in isolation and, when this is performed as in our study, it is clear that there is not an increased hazard ratio at trial start for oral compared with injectable formulations.

Previous work has suggested that receptor occupancies above 50% may be sufficient to prevent relapse. Our findings suggested that higher occupancies were more protective against relapse than lower ones.^48,49^ A previous analysis examined when placebo vs active survival curves separate following discontinuation of oral medication. This found that separation occurred later in studies of cariprazine, and proposed this as related to the compound’s longer half-life.^50^ This earlier study was unable to disambiguate the rate of occupancy change from the role of raw occupancy but its findings are consistent with ours in which absolute occupancy is a key feature in preventing relapse.

### Limitations and Future Work

Although occupancy levels were estimated separately for each formulation, within trials we assumed a common starting occupancy level with identical pharmacokinetics and pharmacodynamics across participants. Given that individuals initially have doses individually titrated based on clinical response, the initial estimate of a similar occupancy across individuals may be unrealistic. Future work collecting positron emission tomography measures of receptor occupancy to directly assess individual-level occupancies during antipsychotic withdrawal would be beneficial here. Other assumptions relate to a linear relationship between occupancy or rate of change and relapse risk. As a result, the finding that occupancy levels are associated with relapse risk might not be robust to different assumptions. It is likely, however, given the magnitude of the *P*-value, that even with considerably different assumptions no significant association would be found between the rate of occupancy change and risk of relapse.

All studies were of an identical antipsychotic and were of a similar duration. Analyses of longer duration trials would be of interest, given that, at the group level, this appears to be associated with a reduced benefit of active treatment over placebo.^13^

There are major individual differences in acute response to antipsychotics,^51^ and it is therefore likely that significant inter-individual differences exist in terms of the effects of antipsychotic withdrawal. Given that specific dopamine antagonist withdrawal strategies are unlikely to substantially affect the risk of relapse, efforts at identifying predictors of relapse may be a better way of minimizing relapse at a group level.^42^

## Conclusion

The risk of relapse is directly related to receptor occupancy and not to the rate of discontinuation of antipsychotics. A clinical question remains as to how to optimally discontinue antipsychotic medication in individuals who request this. The current findings suggest a prolonged discontinuation is unlikely to have a benefit in reducing the risk of relapse. Despite this, a gradual approach may still have benefits in that it may reduce withdrawal symptoms, encourage more regular clinical contact, allow for prompt dose reinstatement if needed, and may allow patients to find a lower dose of medication with a better side effect profile that allows them to continue with treatment.

## Supplementary Material

sbad139_suppl_Supplementary_File
