# Supplementary material for: Does Slow and Steady Win the Race? Rates of Antipsychotic Discontinuation, Antipsychotic Dose, and Risk of Psychotic Relapse
Source: Schizophr Bull. 2023 Oct 5;50(3):513–20. doi: 10.1093/schbul/sbad139 (PMC11059789; doi:10.1093/schbul/sbad139)
Supplement: sbad139_suppl_Supplementary_File [file sbad139_suppl_supplementary_file.docx]

**Supplementary Information**

**Trough plasma level calculation**

C_min_ (the trough plasma level associated with 80% receptor occupanucy) was calculated as follows:

C_min_ = (ED50 x Occ)/(Occ_max_-Occ)

ED50 = 2.38 mg/day

Occ = Target occupancy (e.g. 80%)

Occ_max_ = Maximum possible occupancy (100%)

**Peak plasma level calculation**

C_max_ (the maximum plasma level in the active treatment arm) was calculated as follows:

C_max_ = 1/(0.5^(dosinginterval – t_max_)/c_min_

Dosing interval = Time between dosing (e.g. 30 days for the 1-monthly formulation)

t_max_ = Time until C_max_ from time of injection (e.g. 5 days for the 1-monthly formulation)

**Sensitivity analyses**

*75% occupancy*

In the active treatment arm receptor occupancies vary between 75-82%

**
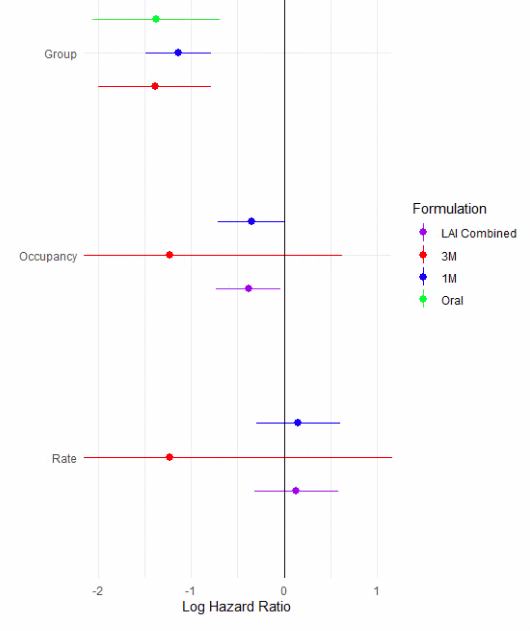
**

*eFigure 1* Hazard ratios relating to randomisation group, receptor occupancy, and rate of occupancy change

For the ‘Group’ set of points a negative log hazard ratio represents a reduced risk of relapse for active compared to placebo treatment. B)For the ‘Rate’ set of points a negative log hazard ratio indicates that slower decline in receptor occupancy is associated with a reduced risk of relapse.

For the ‘Occupancy’ set of points a negative log hazard ratio indicates that higher receptor occupancy is associated with a reduced risk of relapse. Horizontal bars represent the 95% confidence interval.

Higher occupancy was associated with lower risk of relapse (estimate -0.39, SE=0.18, p=0.03)

Rate of occupancy reduction was not associated with risk of relapse (estimate 0.13, SE=0.23, p=0.56)

*85% occupancy*

In the active treatment arm receptor occupancies vary between 85-90%.

**
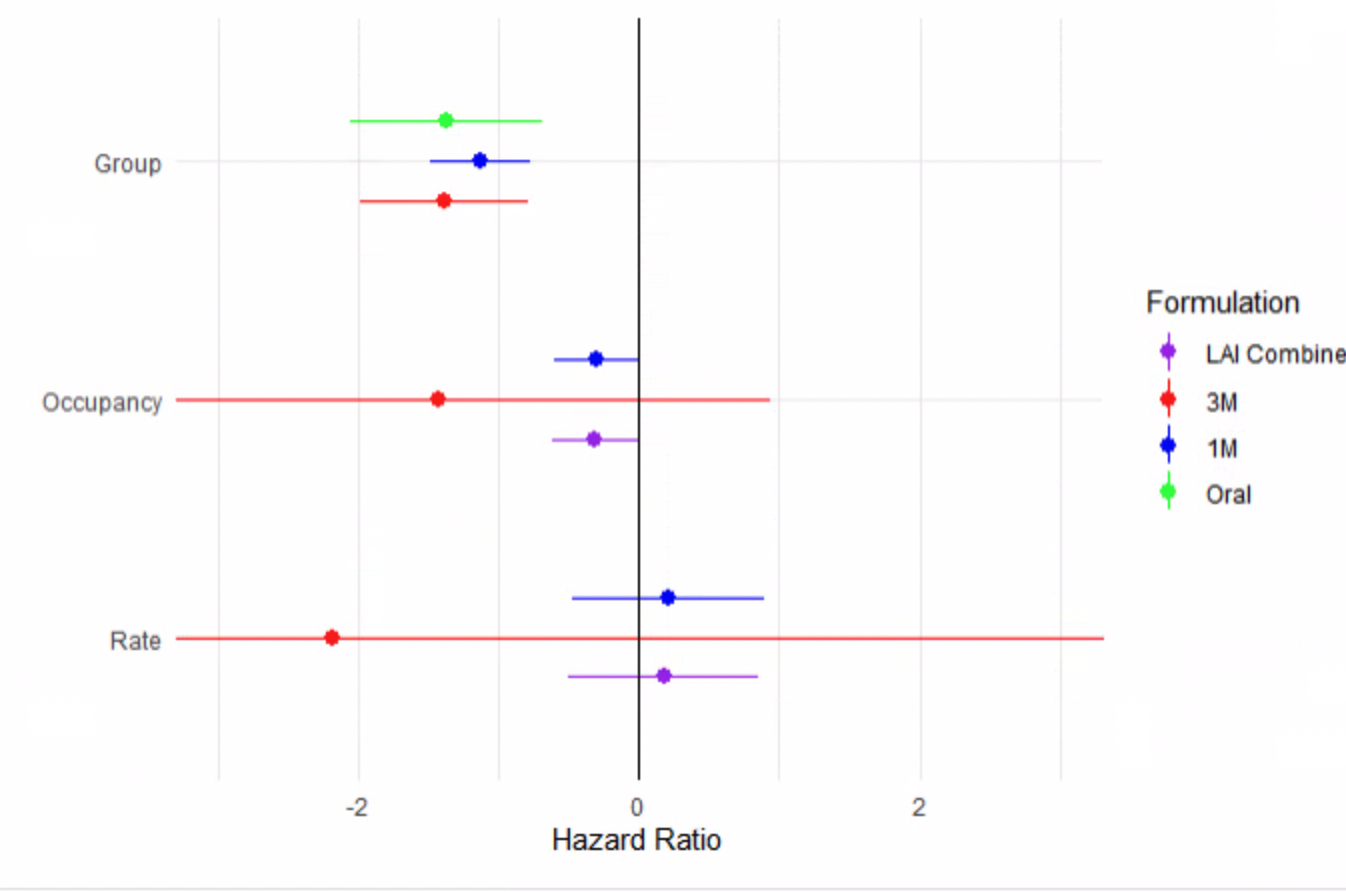
**

*eFigure 2* Hazard ratios relating to randomisation group, receptor occupancy, and rate of occupancy change

For the ‘Group’ set of points a negative log hazard ratio represents a reduced risk of relapse for active compared to placebo treatment. B)For the ‘Rate’ set of points a negative log hazard ratio indicates that slower decline in receptor occupancy is associated with a reduced risk of relapse.

For the ‘Occupancy’ set of points a negative log hazard ratio indicates that higher receptor occupancy is associated with a reduced risk of relapse. Horizontal bars represent the 95% confidence interval.

Higher occupancy was associated with lower risk of relapse (estimate -0.32, SE=0.18, p=0.04)

Rate of occupancy reduction was not associated with risk of relapse (estimate 0.18, SE=0.36, p=0.61)
